# Structural, Electronic and Magnetic Properties of a Few Nanometer-Thick Superconducting NdBa_2_Cu_3_O_7_ Films

**DOI:** 10.3390/nano10040817

**Published:** 2020-04-24

**Authors:** Marco Moretti Sala, Marco Salluzzo, Matteo Minola, Gabriella Maria De Luca, Greta Dellea, Vesna Srot, Yi Wang, Peter A. van Aken, Matthieu Le Tacon, Bernhard Keimer, Claudia Dallera, Lucio Braicovich, Giacomo Ghiringhelli

**Affiliations:** 1 Dipartimento di Fisica, Politecnico di Milano, Piazza Leonardo da Vinci 32, I-20133 Milano, Italy; greta.dellea@gmail.com (G.D.); claudia.dallera@polimi.it (C.D.); lucio.braicovich@polimi.it (L.B.); 2 CNR-SPIN, Complesso Monte Sant’Angelo-Via Cinthia, I-80126 Napoli, Italy; marco.salluzzo@spin.cnr.it (M.S.); gabriellamaria.deluca@unina.it (G.M.D.L.); 3 Max Planck Institut für Festkörperforschung, Heisenbergstrasse 1, D-70569 Stuttgart, Germany; m.minola@fkf.mpg.de (M.M.); V.Srot@fkf.mpg.de (V.S.); y.wang@fkf.mpg.de (Y.W.); p.vanaken@fkf.mpg.de (P.A.v.A.); B.Keimer@fkf.mpg.de (B.K.); 4 Dipartimento di Fisica “E. Pancini”, Università Degli Studi di Napoli “Federico II”, Complesso Monte Sant’Angelo-Via Cinthia, I-80126 Napoli, Italy; 5 Institute for Quantum Materials and Technologies, Karlsruhe Institute of Technology, Hermann-v.-Helmholtz Platz 1, 76344 Eggenstein-Leopoldshafen, Germany; matthieu.tacon@kit.edu; 6 CNR-SPIN, Dipartimento di Fisica, Politecnico di Milano, Piazza Leonardo da Vinci 32, I-20133 Milano, Italy

**Keywords:** unit cell-thick films, superconductivity, resonant inelastic x-ray scattering

## Abstract

Epitaxial films of high critical temperature ($T_{c}$) cuprate superconductors preserve their transport properties even when their thickness is reduced to a few nanometers. However, when approaching the single crystalline unit cell (u.c.) of thickness, $T_{c}$ decreases and eventually, superconductivity is lost. Strain originating from the mismatch with the substrate, electronic reconstruction at the interface and alteration of the chemical composition and of doping can be the cause of such changes. Here, we use resonant inelastic x-ray scattering at the Cu $L_{3}$ edge to study the crystal field and spin excitations of NdBa${}_{2}$Cu${}_{3}$O${}_{7-x}$ ultrathin films grown on SrTiO${}_{3}$, comparing 1, 2 and 80 u.c.-thick samples. We find that even at extremely low thicknesses, the strength of the in-plane superexchange interaction is mostly preserved, with just a slight decrease in the 1 u.c. with respect to the 80 u.c.-thick sample. We also observe spectroscopic signatures for a decrease of the hole-doping at low thickness, consistent with the expansion of the *c*-axis lattice parameter and oxygen deficiency in the chains of the first unit cell, determined by high-resolution transmission microscopy and x-ray diffraction.

## 1. Introduction

The quest for miniaturization of technological devices has pushed the development of nanotechnology, a field of applied research that deals with materials whose properties are intimately related to, and strongly dependent on their lateral size and shape. Ultimately, when the process of miniaturization is taken to the extreme, the resulting material could be made by one or few of its building blocks, which in the case of crystalline solids is the compound unit cell, i.e., the smallest group of atoms that possesses the overall symmetry of the crystal. Within this framework, we investigated the properties of unconventional high-temperature superconductors in the form of films, with variable thicknesses down to the single unit cell, in order to monitor the evolution of their physical properties as a function of thickness and possibly isolate new phenomena.

High-$T_{c}$ superconductors are materials that can have critical superconducting temperatures exceeding that of the boiling point of liquid nitrogen, and mostly include copper- and iron-based materials. They are “unconventional” superconductors, because their properties cannot be accounted for by the conventional Bardeen-Cooper-Schrieffer (BCS) theory of superconductivity [1]. High-temperature superconductivity was discovered in 1986 by Bednorz and Müller in an oxide of Ba, La and Cu [2] and ever since physicists worldwide have been trying to explain its mechanism, which remains to date a subject of intense debate. Here, we focus our attention on ultrathin films of NdBa${}_{2}$Cu${}_{3}$O${}_{7-x}$, the member of a wide class of superconducting copper oxides (Nd,Y)Ba${}_{2}$Cu${}_{3}$O${}_{7-x}$ with critical temperatures above 90 K at ambient pressure. It is known that the transport properties of cuprate epitaxial films tend to degrade at extremely low thicknesses, in particular $T_{c}$ decreases and eventually superconductivity is lost [3,4,5,6]. Despite a quasi-perfect lattice matching with the substrates, that ensures high-quality crystalline growth above few nanometers, at very low coverage epitaxial strain might be imposing a non-negligible deformation to the cuprate structure [7]. Moreover, at the interface the electronic structure might feel the symmetry breaking and undergo some degree of reconstruction. Finally, and even more importantly, the actual chemical composition might be altered, leading to an effective doping of the CuO${}_{2}$ planes different from that of thicker films. A good evidence of the latter effect for the NBCO and YBCO families has been given in the recent literature, based on transport and structural studies [4,6]. What is missing is a spectroscopic analysis. Resonant inelastic x-ray scattering is ideally suited for this purpose, as it probes the crystal field (dd) excitations that are related to local coordination of the Cu ions [8,9,10], and the spin excitations (paramagnons) [11,12,13,14,15], whose dispersion is linked to the in-plane superexchange interaction. The latter is particularly significant for ultrathin films: is the antiferromagnetic short and medium range order preserved also in the single unit cell limit, where only 2 CuO${}_{2}$ planes are present, or is the quasi-perfect two-dimensionality of the system an obstacle to such order? In order to correlate the changes in the crystallographic, electronic and magnetic properties of the films as a function of thickness, we carried out systematic structural and spectroscopic investigations. Note that RIXS measurements on low dimensional materials were reported previously in a superlattice consisting of 25 repetitions of La${}_{2}$CuO${}_{4}$/LaAlO${}_{3}$ building block layers, corresponding to an effective probed thickness of 33 nm [16]. Here, on the contrary, we probe a single layer of cuprate material, thus opening the way to the study of 2D-atomic materials by RIXS down to the single elementary unit cell.

## 2. Materials and Methods

**Figure 1:**
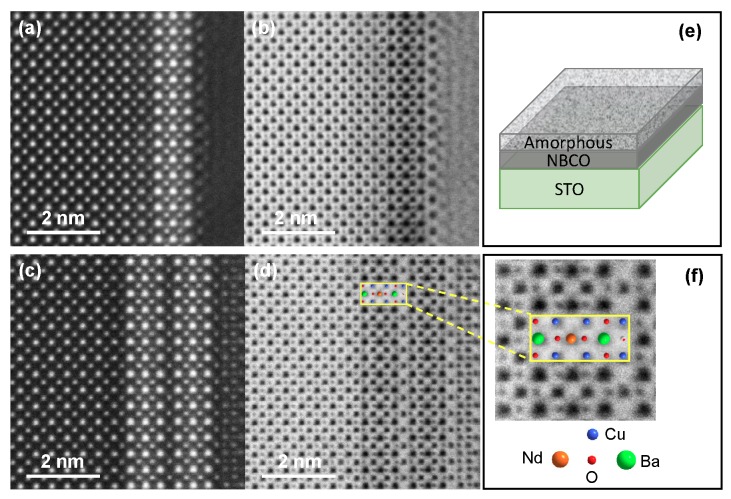
Scanning Transmission Electron Microscopy (STEM) images of epitaxial NBCO ultrathin films grown on STO with an overlay of the NBCO structural model. Images were obtained with the high-angle annular dark-field (HAADF) (panels **a**,**c**) and annular bright-field (ABF) (panels **b**,**d**) techniques. Panels (**a**,**b**) are for the 1 u.c. thick film, panels (**c**,**d**) for the 2 u.c. thick film. The details of atom position assignment is shown in panel (**f**). The scheme of the film growth structure is shown in panel (**e**).

**Figure 2:**
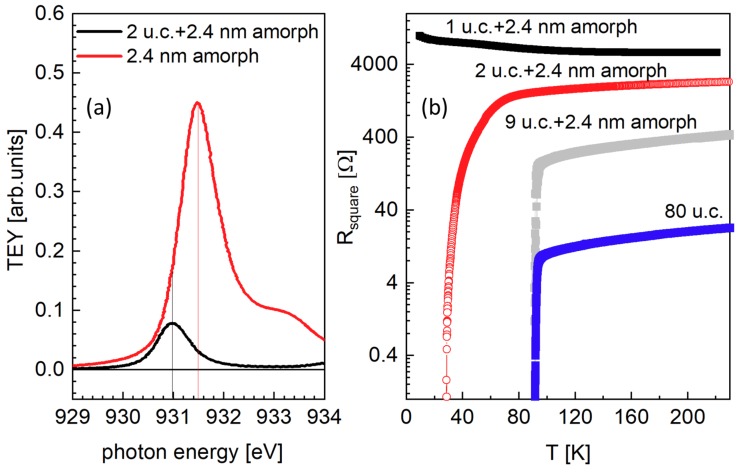
Panel (**a**) XAS measurements in total electron yield (TEY) mode of 2 u.c.-thick NdBa${}_{2}$Cu${}_{3}$O${}_{7-x}$ film covered with a 2.4 nm-thick amorphous layer of the same material (red line) and of the 2.4 nm-thick amorphous layer alone (black line). Panel (**b**) Resistivity measurements of 1 u.c. (black symbols), 2 u.c. (red symbols), 9 u.c. (gray symbols) -thick NdBa${}_{2}$Cu${}_{3}$O${}_{7-x}$ films covered with a 2.4 nm-thick amorphous layer of the same material and of 80 u.c.-thick NdBa${}_{2}$Cu${}_{3}$O${}_{7-x}$ film (blue symbols) with no capping layer.

Films of NdBa${}_{2}$Cu${}_{3}$O${}_{7-x}$ were grown on TiO${}_{2}$-terminated and (001)-oriented SrTiO${}_{3}$ substrates by high pure oxygen pressure diode sputtering [4,6]. In particular, we focus here on the 1, 2 and 80 u.c.-thick films: the growth rate was accurately calibrated by systematic x-ray reflectivity measurements on samples deposited with different deposition time, from 2 to 120 min, and resulted to be 0.5 unit cells per minute. The uncertainty in the film thickness is only half unit cell, as confirmed by high-resolution scanning transmission electron microscopy (STEM) [17,18,19]. The quality of the crystalline structure is shown in the STEM images of Figure 1. In order to preserve the film quality, the epitaxial films were covered with a 2.4 nm-thick amorphous layer of NdBa${}_{2}$Cu${}_{3}$O${}_{7-x}$ with the same nominal stoichiometry of the film, deposited at room temperature, which provides a barrier against external perturbation and prevents change in the oxygen stoichiometry. As shown in Figure 2 (left panel), the X-ray absorption spectra of an amorphous (2.4 nm) NBCO is characterized by a very small absorption due to copper, but does not give any significant contribution to the RIXS measurements at the peak of the Cu $L_{3}$ absorption of the crystalline samples. Transport data shown in Figure 2 (right panel) show that a 1 u.c.-thick film is not superconducting, while superconductivity is observed already in the 2 u.c.-thick sample, with a reduced $T_{c}$ of 30 K, while the maximum $T_{c}$ of 93 K is recovered already in 9 u.c.-thick samples, even when capped with the amorphous layer.

X-ray diffraction (XRD) θ/2θ measurements were performed by a conventional four-circle diffractometer (modified RIKAGU D-Max) equipped with a Cu-x-ray anode source. Transmission electron microscopy (TEM) measurements were carried out at the Stuttgart Center for Electron Microscopy of the Max Planck Institute for Solid State Research in Stuttgart (D). Resonant inelastic x-ray scattering (RIXS) measurements were performed at the ADRESS beam line [20] of the Swiss Light Source at the Paul Scherrer Institute (SLS-PSI) in Villigen (CH) using the SAXES [21] spectrometer. In order to obtain a reasonable count rate also on ultrathin films, the combined energy resolution was set to 190 meV full-width-at-half-maximum at 931 eV, corresponding to the main peak in the Cu $L_{3}$ edge x-ray absorption (XAS) profile of crystalline NdBa${}_{2}$Cu${}_{3}$O${}_{7-x}$. The polarization of the incoming photons was horizontal in the laboratory reference frame and parallel to the scattering plane. The scattering angle was set to 130${}^{\circ }$ in order to maximize the available momentum transfer. All measurements were performed at T=10 K, so that possible radiation damage effects were minimized.

## 3. Results and Discussion

NdBa${}_{2}$Cu${}_{3}$O${}_{7}$ in bulk form has an orthorhombic crystal structure with lattice parameters a=3.87 Å, b=3.91 Å and c=11.74 Å and with CuO chains running along the *b*-axis separating bilayer units of CuO${}_{2}$ planes. XRD measurements show that above a threshold film thickness of about 60 u.c. the NdBa${}_{2}$Cu${}_{3}$O${}_{7-x}$ film is fully relaxed and its lattice parameters match those of bulk samples. Below 60 u.c., instead, NdBa${}_{2}$Cu${}_{3}$O${}_{7-x}$ films are subject to a small in-plane tensile strain and adopt a tetragonal crystal structure with in-plane lattice parameters perfectly matched with the STO substrate (a=b=3.905 Å) and short CuO chains running along both a and b axis. This suggests a clean pseudomorphic growth during the epitaxial deposition. Nevertheless, despite the sign of the strain, the *c*-axis increases as the film thickness decreases. For samples capped with the amorphous NBCO, the maximum value of the *c*-axis is 11.80 Å in 1 u.c.-thick films. The combination of transport and x-ray diffraction results suggests a direct correlation between the *c* lattice parameter and the doping level of NdBa${}_{2}$Cu${}_{3}$O${}_{7-x}$: in particular, as for other high-T${}_{c}$ cuprates of the so-called 123-family, the *c*-axis expands when the oxygen stoichiometry in the chains decreases. Thus, the *c*-axis is one-to-one related to the oxygen stoichiometry and to the effective doping level of NdBa${}_{2}$Cu${}_{3}$O${}_{7-x}$. In our case, we estimate x=0.6, 0.5 and about 0 for 1, 2 and 80 u.c.-thick films, respectively, consistent with transport measurements and with the chemical analysis by high-resolution electron microscopy [17,19].

**Figure 3:**
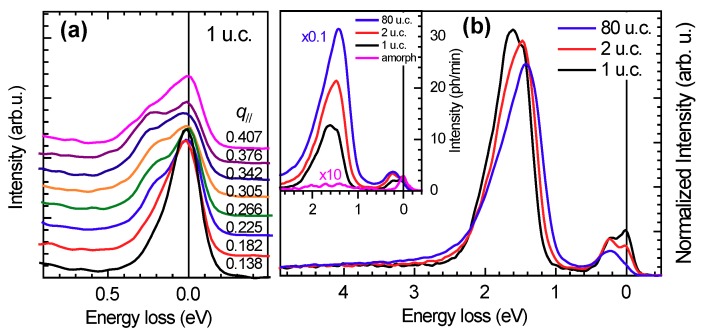
Panel (**a**) RIXS measurements of magnetic excitations in 1 u.c.-thick NdBa${}_{2}$Cu${}_{3}$O${}_{7-x}$ film as a function of momentum transfer $q_{\|}$. Panel (**b**) Comparison between the RIXS spectra at $q_{\|}=0.376$ r.l.u. of 1, 2 and 80 u.c.-thick NdBa${}_{2}$Cu${}_{3}$O${}_{7-x}$ films after normalization to the area of the dd excitations. Also shown is the RIXS spectrum of a 2.4 nm-thick amorphous layer of NdBa${}_{2}$Cu${}_{3}$O${}_{7-x}$, rescaled by a factor 10. The inset shows the same data normalized to the acquisition time.

The Figure 3**b** shows representative RIXS spectra of 1, 2 and 80 u.c.-thick films at momentum transfer $q_{\|}=0.376$ r.l.u.. The acquisition time was 8 minutes for the 80 u.c.-thick sample and 30 min for the thinner ones, but data were normalized to the area of the spectrum in the energy region between 1 and 3 eV energy loss because of the large absolute count-rate variation between the various samples. However, we first of all would like to emphasise the strong light-matter interaction that characterises RIXS, as opposed to the weak neutron-matter interaction in inelastic neutron scattering (INS), which makes RIXS suitable to study electronic, including magnetic, excitations in layers with thicknesses down to the single unit cell, in a reasonable amount of time.

The spectra in Figure 3 are characterized by a quasi-elastic line at zero energy loss, spin excitations around 0.3 eV [22], while the most intense excitations at around 1.5 eV are crystal-field (or dd) transitions [10,21,22]. The inset in the same figure shows that the contribution of the amorphous NdBa${}_{2}$Cu${}_{3}$O${}_{7}$ protective layer to the RIXS signal is negligible. Indeed, the XAS spectra of crystalline and amorphous NdBa${}_{2}$Cu${}_{3}$O${}_{7}$ show absorption resonances at distinct energies: this difference provides chemical sensitivity to RIXS and can be used to distinguish the contribution of Cu ions in different chemical environments.

First, we discuss the effect of the film thickness on dd excitations, i.e., transitions between the crystal field split states of Cu${}^{2+}$. For bulk, insulating NdBa${}_{2}$Cu${}_{3}$O${}_{6}$, past RIXS studies allowed to determine with precision the following sequence of transition energies: the $3z^{2}-r^{2}$, xy and zx/yz states of the 3$d^{9}$ hole are found at 1.98, 1.52 and 1.75 eV, respectively [10]. In the presence of doping, however, the corresponding peaks broaden and merge in an asymmetric, triangular feature, making the assignment of each individual final state impossible. The shape of the crystal-field excitation peak, therefore, reflects the degree of doping in the system: in our case, we observe a gradual evolution of its shape from very broad and asymmetric to rather sharp and symmetric as the film thickness is reduced, consistent with the variation of effective doping level invoked before.

**Figure 4:**
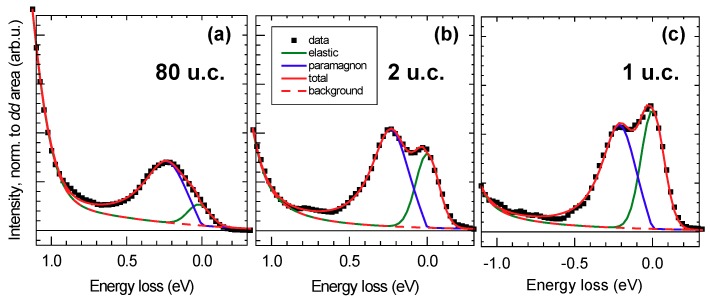
Panels (**a**–**c**): Fit to the RIXS spectra of 80, 2 and 1 u.c-thick NdBa${}_{2}$Cu${}_{3}$O${}_{7-x}$ films at $q_{\|}=0.376$ r.l.u.

We now turn to the analysis of the magnetic excitations, which in superconducting cuprates are heavily damped spin-excitations in the absence of long-range magnetic ordering and are, therefore, named paramagnons. Figure 3a shows that magnetic excitations disperse as a function of the momentum transfer $q_{\|}$. The low-energy region of the RIXS spectra was fitted with two curves, an energy-resolution limited Gaussian for the elastic line and an asymmetric Voigt function for the paramagnon. Details of the fit for $q_{\|}=0.376$ r.l.u. are reproduced in Figure 4a–c. We note that the elastic line intensity increases as the film thickness is reduced, thus suggesting a growing non-resonant elastic scattering contribution from the substrate.

**Figure 5:**
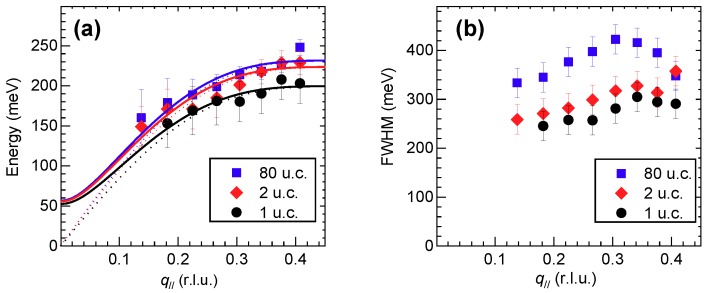
(**a**) Dispersion and (**b**) FWHM of magnetic excitations in 1 (black circles), 2 (red diamonds) and 80 (blue squares) u.c.-thick NdBa${}_{2}$Cu${}_{3}$O${}_{7-x}$ films. Continuous (dotted) lines show the corresponding optical (acoustic) magnetic branch used to fit the dispersion.

A summary of the main fitting parameters used to fit the RIXS data summarized in Figure 3 is provided in Figure 5. In particular, Figure 5a shows that the paramagnon energy slightly diminishes with the film thickness: the experimental data points are well fitted to the dispersion curve of optical magnons within the framework of a simple Heisenberg model, which provides the main in-plane (and out-of-plane) magnetic coupling(s) $J_{\|}$ (and $J_{⊥}$, respectively). Note that, despite few authors have adopted a different interpretation of the spin excitations in doped cuprates [23], this approach is fully justified by a number of previous studies showing that, although damped, the spin excitations in doped cuprates remain well defined, preserve their spectral weight up to very high doping levels beyond optimal and perfectly reproduce the magnon dispersion observed in the AF insulators [12,13,24]. We find that $J_{\|}$ evolves from 98 meV for the 1 u.c.-thick film, to 110 meV for the 2 u.c., to 114 meV for the 80 u.c.-thick film (the out of plane coupling has been fixed at 7 meV as in Ref. [25]). This little change can be consistent with the small variation of the in-plane lattice parameters vs. film thickness. On the other hand, Figure 5b evidences a large variation of the paramagnon width at all transferred momenta: in particular, it is drastically reduced in the thinnest films as compared to the 80 u.c.-thick one. Again, this observation is consistent with the thickness dependence of the effective doping level and, in particular, with the evidence coming from transport measurements that the 1 u.c.-thick film is the least-doped sample. We note that in the data presented here, the mild tensile strain is playing only a secondary role in the determination of the thickness dependence of the crystal field and spin excitation energy. In other cases, e.g., for CaCuO${}_{2}$ [26] and La${}_{2}$CuO${}_{4}$ grown on different substrates [27], the larger strain leads to sizable changes of the hopping integrals that govern the 3d orbital energy splitting and the superexchange parameter $J_{\|}$. On the contrary, here the dominant effect is the oxygen deficiency and correspondingly lower doping level of the ultrathin films, which leads to modifications of the RIXS spectra similar to those observed in bulk crystals, i.e., the broadening of the magnetic peak, with no or little variation of its energy position as a function of doping [28].

## 4. Conclusions

We were able to measure the RIXS response of NdBa${}_{2}$Cu${}_{3}$O${}_{7-x}$ films, with thicknesses down to the single unit cell, i.e., the smallest fundamental constituent of matter in crystalline form. This very fact proves the extreme sensitivity of RIXS in the study of nanometer-sized materials. Specifically, our measurements probe both magnetic and crystal-field (or dd) excitations and provide evidence of stoichiometry variations vs. thickness in nominally chemically equivalent films: in particular, we find that the effective doping of the NdBa${}_{2}$Cu${}_{3}$O${}_{7-x}$ films decreases as the film thickness is reduced. Our results are consistent with previous transport and structural investigations, thus providing a coherent picture of the electronic properties of ultrathin NdBa${}_{2}$Cu${}_{3}$O${}_{7-x}$ films. These results suggest that in cuprates, the degradation of superconducting properties when approaching the single unit cell thickness limit is related to the actual doping at the interface with the substrate rather than to the modification of the Mott-Hubbard physics in the CuO${}_{2}$ planes. Namely, the realization of almost ideal 2D superconductivity is not impeded by the supposed disruption of the strong antiferromagnetic background in the planes. On the contrary, short and medium range magnetic correlation persists down to 1 unit cell, leaving space for the Cooper pairing mediated by spin-fluctuation to be at play also in the extreme case of a single CuO${}_{2}$ plane [29], a scenario realized with half u.c.-thick Bi${}_{2}$Sr${}_{2}$CaCu${}_{2}$O${}_{8+x}$ [30] and recently with Bi${}_{2}$Sr${}_{2}$CaCu${}_{2}$O${}_{8+\delta }$ in the form of 1 unit cell thick flakes [31].
